# Lithium Sulfur Primary Battery with Super High Energy Density: Based on the Cauliflower-like Structured C/S Cathode

**DOI:** 10.1038/srep14949

**Published:** 2015-10-12

**Authors:** Yiwen Ma, Hongzhang Zhang, Baoshan Wu, Meiri Wang, Xianfeng Li, Huamin Zhang

**Affiliations:** 1 Division of Energy Storage, Dalian Institute of Chemical Physics, Chinese Academy of Sciences, 457 Zhongshan Road, Dalian 116023, P.R. China; 2 Collaborative Innovation Center of Chemistry for Energy Materials (iChEM), Dalian 116023, P.R. China

## Abstract

The lithium-sulfur primary batteries, as seldom reported in the previous literatures, were developed in this work. In order to maximize its practical energy density, a novel cauliflower-like hierarchical porous C/S cathode was designed, for facilitating the lithium-ions transport and sulfur accommodation. This kind of cathode could release about 1300 mAh g^−1^ (S) capacity at sulfur loading of 6 ~ 14 mg cm^−2^, and showed excellent shelf stability during a month test at room temperature. As a result, the assembled Li-S soft package battery achieved an energy density of 504 Wh kg^−1^ (654 Wh L^−1^), which was the highest value ever reported to the best of our knowledge. This work might arouse the interests on developing primary Li-S batteries, with great potential for practical application.

Efforts to develop primary batteries with high energy density and low cost have never been stopped ever since the invention of Volt batteries in 18^th^ century^1^,^2^. Many of the significant advances were made during the 1970–90 s period and were promoted by the concurrent development of electronic technology, the new demands for portable power sources and the support for the space, military and the environment improvement programs. Especially in recent years, a large amount of batteries with high energy density have been developed, such as the Zn-O_2_, Mg-O_2_, Al-O_2_, Na-O_2_ and Li-O_2_ systems^3^, which were favorable in oxygen environment. Besides that, batteries within isolated conditions, such as the currently developed Li-SO_2_, Li-SOCl_2_, Li-MnO_2_ and Li-CF_x_ systems, have also been developed for applications such as ocean, out space and etc.

As shown in Table 1^3^,^4^,^5^,^6^,^7^,^8^,^9^,^10^,^11^,^12^,^13^, these primary batteries in development have already achieved high practical specific capacity density, excellent low-temperature performance or high power density. However, there still exists some characteristic problems, such as the unsatisfying security, the voltage hysteresis, the considerable electrochemical polarization or the relatively high material cost. Besides that, increases in the energy density of primary batteries has tapered off during the past decade, as the existing battery systems have matured and the development of new higher energy batteries is limited by the lack of new and/or untried battery materials and chemistries. Therefore, it is still a desirable but challenging task to develop novel primary batteries with high energy density.

Currently, the Li-S batteries attracted great attention throughout the world^14^,^15^,^16^,^17^,^18^,^19^. This battery system possesses extremely low cost, extremely high specific energy density (2600 Wh kg^−1^) and environmental friendliness, rendering it as one of the most promising ones ever developed^20^,^21^,^22^,^23^,^24^,^25^,^26^,^27^,^28^,^29^,^30^,^31^. However, the Li-S battery is usually considered as the secondary batteries, while its great potential as the primary ones has been ignored to some extent. Actually, the cycling stability of Li-S batteries is not satisfying yet compared with the commercial Li-ions ones, but its initial discharge specific capacity is super high, even reaching the theoretical value^32^,^33^, which render it suitable for primary application. For another thing, more theoretical and practical experience could also be accumulated for the secondary Li-S batteries during the development of primary ones. Meanwhile, the rechargeable nature of Li-S system could make it more competitive than the previously reported primary batteries^34^. Currently, a most challenging task for developing Li-S primary battery is to further increase its practical energy density, for competing with other commercial battery systems.

In order to achieve this goal, to design sulfur cathodes with high sulfur content and high sulfur loading (“two high”^35^) are quite essential according to the practical batteries designation^36^. However to increase the utilization of sulfur (or active material) is a challenging issue, due to the increased ohmic resistance or charge-transfer polarization^37^. Given this, the porous structure of the whole cathodes (other than only C/S materials) should be further optimized, for improving the Li-ions transportation, sulfur distribution and accommodation status^26^,^38^,^39^,^40^. However due to the insulation nature of sulfur particles, a large amount of electronic conducting materials (eg. carbon) have to be added in the cathodes^23^. Therefore although great efforts have been engaged in developing high performance C/S composition, the “two high” cathodes were still seldom reported. Although the graphene sponges-S^36^ and CNT-S paper^41^ based cathode could afford sulfur loading up to 10 and 6 mg cm^−2^ separately, further technical innovation is yet needed for assembling them into winding or laminating types based on commercial consideration.

Herein, we reported a kind of Li-S primary battery assembled with unique cauliflower-like structured cathodes. These cathodes own enormous hybrid macro-, meso- and micro-pores, with 75% sulfur content in C/S composite and 6 ~ 14 mg cm^−2^ sulfur loading on aluminum foil. The whole Li-S primary battery with capacity of 6570 mAh was assembled in soft package type with the prepared cathode. As a result, the specific capacity output of this cathode achieved about 1300 mAh g^−1^ (S), with the overall energy density of 504 Wh kg^−1^ (654 Wh L^−1^). This value, to the best of our knowledge, is the highest ever reported in literatures. The detailed preparation process and characterization are discussed as follows.

Figure 1 illustrated the preparation process of the Cauliflower-like 3D connected hierarchical macro-, meso- and micro- porous cathode. First, the KB 600 (KB for short) powder with a large content of meso- and micro-pores was mixed with gelatin, forming particle to particle networks with diameter of 1 ~ 5 μm (Fig. 2a)^42^. The as prepared KB/gelatin network was then carbonated under 900 °C to ensure the electrical conductivity and joint strength between KB single particles in Ar. The gelatin modified KB networks (MKB for short) were then impregnated with elemental sulfur and form the MKB-S, with the sulfur content of 75 wt%^28^. For comparison, the Super P and KB carbon powder was also impregnated with the same sulfur content, named as Super P-S and KB-S respectively. Finally, the C/S composite were mixed with binder and casted onto the aluminum foil with the ratio of 9:1 (m_c/s_ : m_binder_) on a coating machine (Figure S3). The detailed preparation process was illustrated in the supporting information (Figure S1-S2).

The morphology of the as prepared carbon and C/S composite materials was observed under scanning electronic microscope (SEM). As shown in Fig. 2a, the KB particles show irregular aggregation of carbon powder, with primary particle size of 25 nm (Figure S5). After sulfur infiltration, the KB-S particles accumulated to 100 nm rice-like particles caused by the binder nature of sulfur (Fig. 2b). In composition, the MKB shows larger clusters about 1–5 μm jointed by the carbonated gelatin (Fig. 2c). Besides that, the morphology of MKB-S was almost unchanged after sulfur infiltration, which showing good mechanical strength (Fig. 2d).

The porous structure of the carbon particles was further characterized with the nitrogen adsorption/desorption test via the BET method. As shown in Table 2 and Figure S6, the volume of pores >20 nm was obviously increased from 1.23 to 1.96 cm^3^g^−1^, with its percentage in total pore volume increased from 47% to 63%. As is known, the carbonated gelatin is so less that it seems impossible to make such a big difference in porous distribution. Beyond that, the morphology of single carbon particle was also almost unchanged after gelatin carbonation (Figure S5). Then it could be concluded that the increased pore volume (>20 nm) was mainly caused by the accumulation of purposely pre-jointed carbon particle clusters. This part of pores could facilitate the electrolyte infiltration and Li-ions transport across the cathode.

At the same time, the MKB-S is also easier to adhere onto the aluminum foil than the KB-S with the same binder content. As shown in Fig. 3a, the MKB-S cathode showed excellent structural integrity under a certain degree of bending. On the contrary, the KB-S cathodes showed severe dregs and dropped off the aluminum foil (Fig. 3b). This could also be explained by the structural difference between them. As for MKB-S, the single carbon particle has already been pre-linked with carbonated gelatin, so less glue is needed to bond each carbon particles together. At the same time, more glue could be used to bond the carbon particles onto the aluminum foils, achieving excellent mechanical quality. As for the KB-S, however, much higher binder ratio is needed in order to reach the same binder strength^43^. It can be concluded that carbonated gelatin plays a vital role in cohering the carbon particles, which means that our structural design of carbon congeries is of great practical importance.

The surface morphology of MKB-S cathode was further observed under 5 × 10^3^, 5 × 10^4^, 2 × 10^5^ magnitude with SEM. As shown in Fig. 4, the micrometer-size clusters of MKB-S were well reserved in the cathode and formed the cauliflower-like structure. The MKB-S clusters were close-packed randomly on the aluminum foil, setting up a large quantity of interconnected pores in sub-micrometer scale. Meanwhile, enormous meso- and micro- pores were also existed in the single KB particle, further offering large surface area and pore volume for polysulfide species accommodation.

Due to the rich porous structure of the cauliflower cathode, its electrolyte uptake ability was also outstanding. As shown in Fig. 5, the cauliflower-like structured MKB-S cathode showed higher electrolyte uptake per cathode volume or sulfur mass, as compared with the Super P-S cathode. High electrolyte absorption could enhance the sulfur utilization during discharge^44^,^45^,^46^,^47^, so better performance is anticipated for MKB-S cathode. The testing process and calculating method is illustrated in supporting information (section 2.3).

The Li-S performance of the cathodes with sulfur loading ranging from 6 to 14 mg cm^−2^ were tested with coin cells. As shown in Fig. 6, the specific discharge capacity of the cathode with sulfur loading of 6 mg cm^−2^ reached 1310 mAh g^−1^ (S) in the 1^st^ cycle, and remained 746 mAh g^−1^ after 50 cycles (Figure S9). The initial discharge specific capacity reached to 1316 mAh g^−1^ with sulfur loading of 10 mg cm^−2^, and reserved 1040 mAh g^−1^ after 10 cycles (Figure S9). Excitingly, the initial discharge specific capacity still reached to 1310 mAh g^−1^ when the sulfur loading increased to 14 mg cm^−2^. The high specific capacity and excellent cycling performances of the “two high” cathodes should be ascribed to the unique 3D structure. On the one hand, the numerous meso- and micro- pores in the cauliflower-like structure enhanced the utilization and accommodation of sulfur species. On the other hand, the channels among the cauliflower clusters (in micrometer scale) could facilitate the penetration of electrolyte into all MKB-S composite, which further ensured the penetration of electrolyte throughout each carbon spheres. In comparison, however, the randomly casted Super P-S based cathodes could only deliver specific capacity of about 900 mAh g^−1^ (S), due to the relatively low meso-/micro- pore volume and low electrolyte uptake in accordance with the previously reports^48^. To put in a nut shell, the as designed cauliflower-like structure ensured the free transportation of Li-ions and the high utilization of sulfur particles, rendering it possible to achieve super high practical energy density.

To further analyze the structure-performance relationship of the cauliflower-like MKB-S cathodes, the surface morphology change before and after discharge was observed. Due to the difficulty to prepare high quality cathodes using KB-S materials, the cathodes made from the most studied Super P-S composite was chosen for comparison. Figure 7 shows the SEM images of Super P-S and MKB-S cathodes before and after battery discharge. It was clearly observed that the pristine Super P-S cathodes exhibited a monolithic structure in contrast to the “cauliflower-like” morphology of MKB-S. After discharging, much more Li_2_S or Li_2_S_2_ were produced inner the MKB-S cathodes than the Super P-S cathodes, due to the fact that much higher discharge capacity was achieved by MKB-S cathode (Fig. 6a,b). As observed from Fig. 7b,d, however, the Super P-S cathodes were totally covered by a layer of Li_2_S/Li_2_S_2_ precipitates while the MKB-S cathodes remained the interconnected porous structure. This means that the cauliflower-like MKB-S structure provided enough volume and surface area to accommodate the Li_2_S/Li_2_S_2_ deposition. Besides that, the Li-ions transportation channels were also reserved during discharge, thus ensure a higher capacity discharge. By the way, although the macro-pores in MKB-S seemed increased, it should be ascribed to the redistribution of sulfur species inner the carbon particles.

Figure 8 could explain the structure-performance relationship of both cathodes from a macroscopic view. During the discharge process, partial polysulfide would be released into the bulk electrolyte and redeposit onto the carbon surface^49^. And the polysulfide are prone to be reduced on the cathode surface near the separator due to the shortest Li-ions transport path^39^. As a result, the discharge products of Li_2_S and Li_2_S_2_ would easily cover the electrolyte surface, especially for the Super P cathode which owns narrower intervals among carbon particles. As for the MKB cathode, however, the much larger intervals (in micrometer scale) is hard to be blocked, which ensures the Li-ions conductivity.

Considering the commercial application issues in future, Li-S batteries with low self-discharge rate (or long shelf life) are also required. Due to the high reactivity of lithium and sulfur species, however, vice reaction near the lithium interface is always prone to take place. After a long resting time, this might result in a significant decrease of the open-circuit voltage and even the disappearance of the first discharge plateau (around 2.3 V)^50^. Excitingly, the self discharge problem could be solved by forming stable SEI with LiNO_3_ additive, without worrying about its exhaustion because that only several discharging procedure is needed for the primary battery application. As a result in our lab, the MKB-S cathodes exhibited no capacity decay even after a resting time of 30 days at room temperature. As shown in Fig. 9, the specific capacity for the initial discharging process is still over 1300 mAh g^−1^, which is similar to that before shelf testing. Furthermore, the upper discharge plateau around 2.3 V and a lower discharge plateau around 2.1 V increased slightly due to the improved electrolyte infiltration. It should also be noted that, the battery could still cycle for another tens of times (Figure S11), which could be of great value for some special application such as the weapons. Besides LiNO_3_, other technological improvements of separators or electrolytes could also help to improve the shelf stability.

For ultimately testing the practical performance of the as designed cauliflower-like MKB-S cathodes, the Li-S soft package batteries were assembled in stack type^51^. The sulfur loading in each cathode was above 6 mg cm^−2^. As shown in Fig. 10, the discharge specific capacity and energy density reach 1304 mAh g^−1^ and 504 Wh kg^−1^ (654 Wh L^−1^), respectively. This value, to the best of our knowledge, is the highest ever reported in literatures and is quite competitive to other commercial primary batteries. Due to its much higher theoretical energy density, the Li-S primary battery system could achieve higher energy density, showing great potential for commercialization in future^26^.

In conclusion, the Li-S system is quite promising as primary batteries, which however has been ignored for a long period. The as designed Li-S battery could achieve super high energy density and good shelf stability, let alone the excellent low cost and environmental friendly characters, rendering it promising for commercial application in future. Currently, much more research work is needed for further increasing the energy density as well as the safety of Li-S primary batteries.

## Conclusion

Primary battery with lithium and sulfur as the active materials were studied in this article, which achieved energy density of 504 Wh kg^−1^ (654 Wh L^−1^) using a self-designed cauliflower-like cathode for the first time. Due to the hierarchical macro-, meso- and micro-pores in the cathode, a specific capacity about 1300 mAh g^−1^ (S) was achieved with sulfur loading of 6 ~ 14 mg cm^−2^. Furthermore, the as designed primary battery shows extremely low self-discharge rate, with almost no capacity decay during a month. More importantly, due to the fact that the Li-S battery could also be recharged for several times, it is appealing for practical utilization. This research work is considered to arouse the interests of primary Li-S batteries, shedding light on the development of Li-S batteries.

## Methods

### Material preparation

The preparation of modified Kejent black (MKB) carbon clusters were prepared as follows. First, 4 g Kejent black (KB) was blend with 1g gelatin in much water, and then dried at 60 °C to get the KB-gelatin composite. Then the composite was carbonated at 900 °C to get MKB. The KB and Super P carbon particles were directly used after received. The carbon/sulfur (C/S) composite (MKB-S, KB-S and Super P-S) was prepared by incorporating the sublimed sulfur into the porous carbon via melt-diffusion method at 155 °C for 4 h in argon filled vessel. For both samples, the sulfur content was 75 wt%. The cathode was prepared with by casting the C/S composites on to aluminum foil, with the content of C/S: binder of 9:1. The sulfur loading on aluminum foil was controlled to be 6, 10 and 14 mg cm^−2^. The more detailed material preparation process was illustrated in supporting information.

#### Materials characterizations

Brunauer–Emmett–Teller (BET) method was used for the surface area measurement. Barrett–Joyner–Halenda (BJH) adsorption-desorption was used for the pore analysis. Morphology of the samples was characterized by Transmission Electron Microscope (TEM, JEM-2100) and Scanning Electron Microscope (SEM, QUANTA 200 FEG and JSM-7800F). The electrolyte uptake and effective porous data of cathodes were tested with TEGDME as the soaking medium.

#### Battery evaluations

Coin cells of 2016 type were assembled in an Argon-filled glove box with lithium foil as the anode, Cellgard 2325 as the separator and 1.5 M LiTFSI in DME/DOL (1:1 v/v, with 5 wt% LiNO_3_) as the electrolyte. Soft package type batteries were assembled in dry-room with the cathode, separator and anode in stack type. The capacity of the battery was about 10 Ah. The charge-discharge test was carried out using LAND CT-2001A equipment (Wuhan Landian Corp.) and Arbin (BT2000, MIT), and the cut-off potential for charge and discharge was set to 2.8 V and 1.7 V, with discharge current of 0.01C (1 C = 1675 mAh g^−1^) respectively.

## Additional Information

**How to cite this article**: Ma, Y. *et al.* Lithium Sulfur Primary Battery with Super High Energy Density: Based on the Cauliflower-like Structured C/S Cathode. *Sci. Rep.* **5**, 14949; doi: 10.1038/srep14949 (2015).

## Supplementary Material

Supplementary Information

Supplementary Video S1

## Figures and Tables

**Figure 1:**
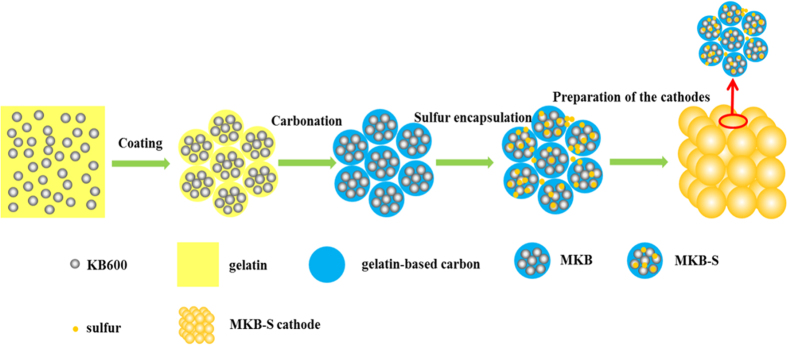
Schematic illustration of synthesis steps of the cauliflower-like electrode.

**Figure 2:**
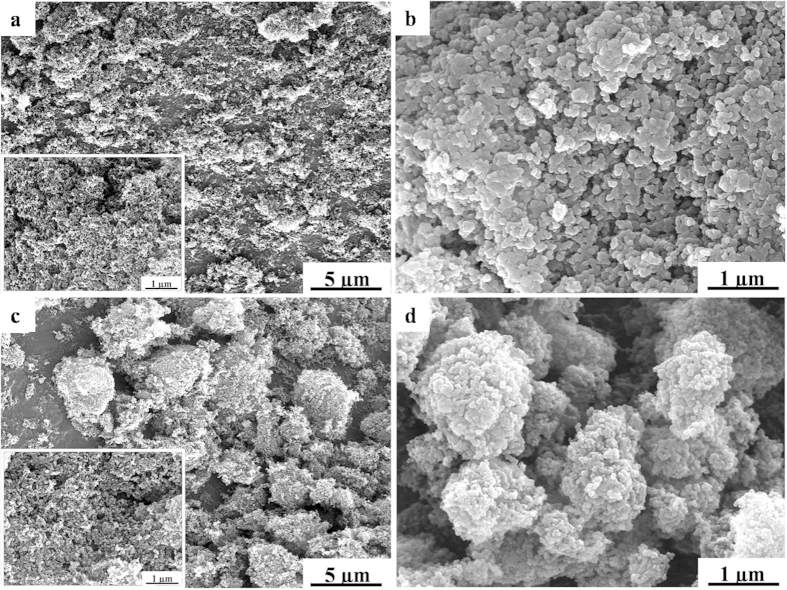
FESEM images of (a) KB, (b) KB-S, (c) MKB, (d) MKB-S.

**Figure 3:**
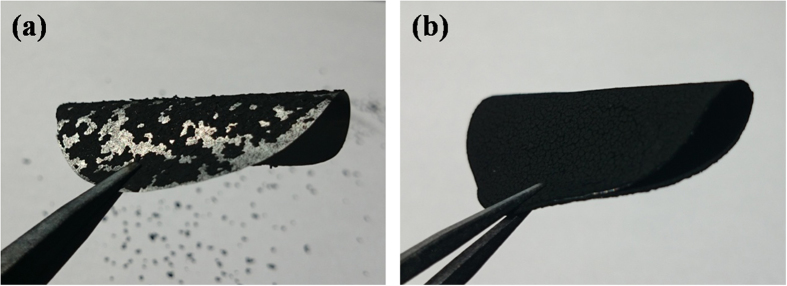
Photograph of the (a) KB-S and (b) MKB-S cathodes.

**Figure 4:**
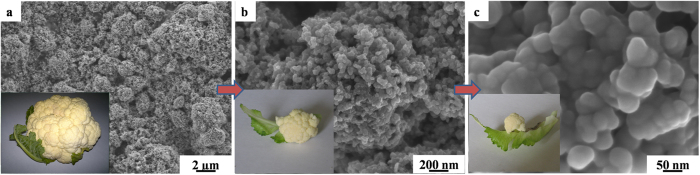
SEM images of MKB: (a) 5 × 10^3^ magnitude, (b) 5 × 10^4^ magnification, (c) 2 × 10^5^ magnitude, with the insert photography of corresponding section of a cauliflower.

**Figure 5:**
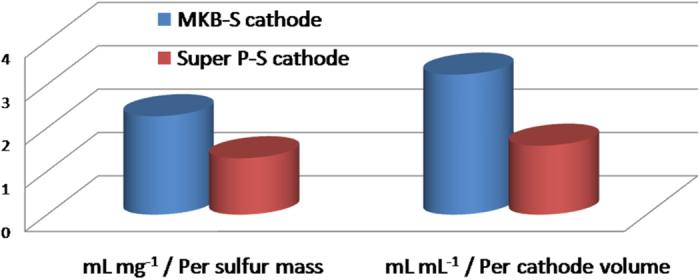
Elelctrolyte uptake of the cathodes (with date from Table S1.

**Figure 6:**
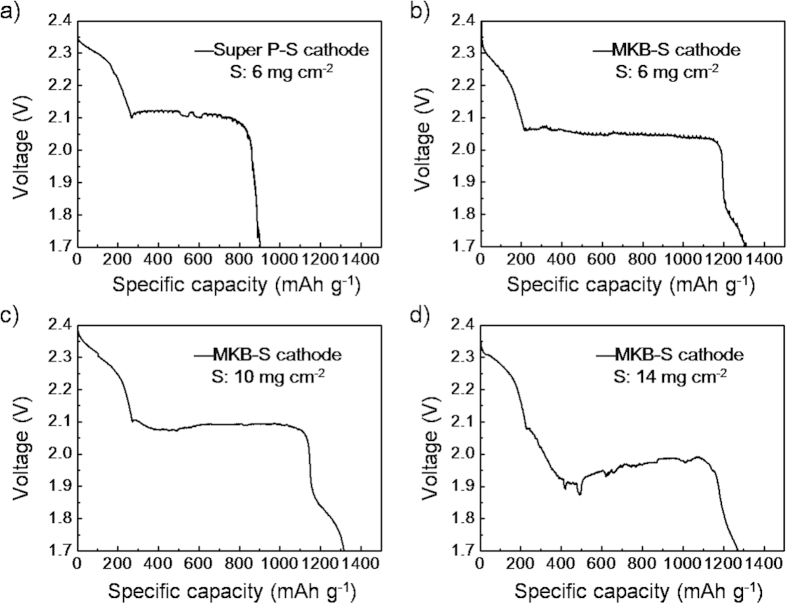
The discharge voltage profiles of the 1^st^ cycle for the Super P-S cathode with sulfur loading of (a) 6 mg cm^−2^, and MKB-S cathode with sulfur loading of (b) 6 mg cm^−2^, (c) 10 mg cm^−2^ and (d) 14 mg cm^−2^, respectively.

**Figure 7:**
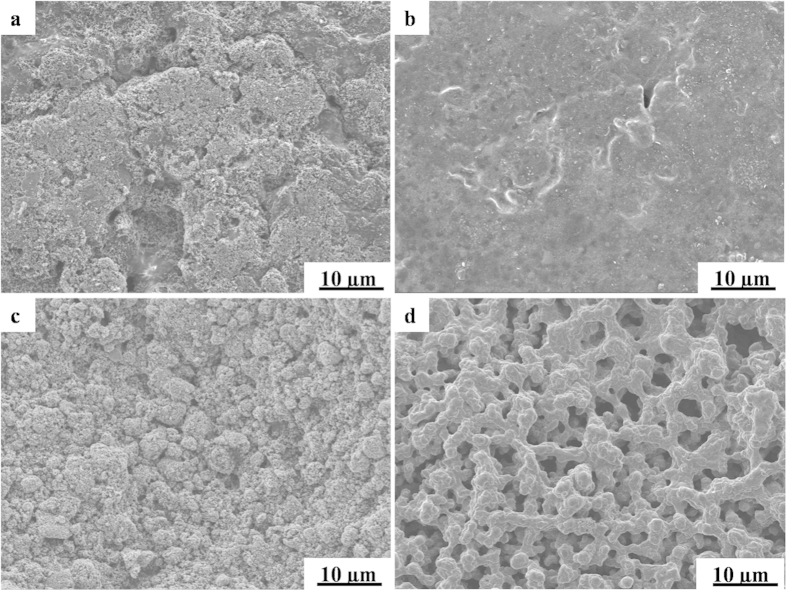
Schematic diagrams of morphologies of MKB cathode and Super P cathode. (**a**,**c**) original and (**b**,**d**) after discharging.

**Figure 8:**
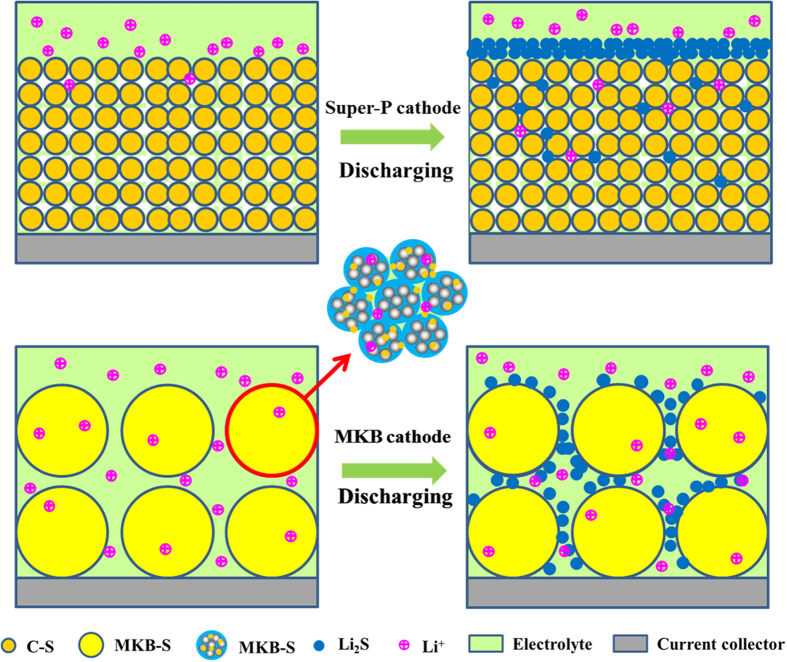
Schematic diagrams of morphologies of MKB-S cathode and Super P-S cathode. (**a**,**c**) original and (**b**,**d**) after discharging.

**Figure 9:**
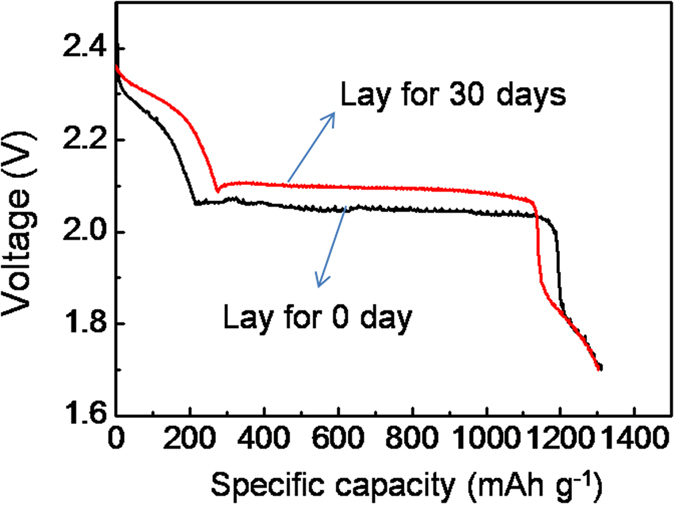
The discharge voltage profiles for the MKB-S cathode with different resting time. The sulfur loading in the cathode is 6 mg cm^−2^.

**Figure 10:**
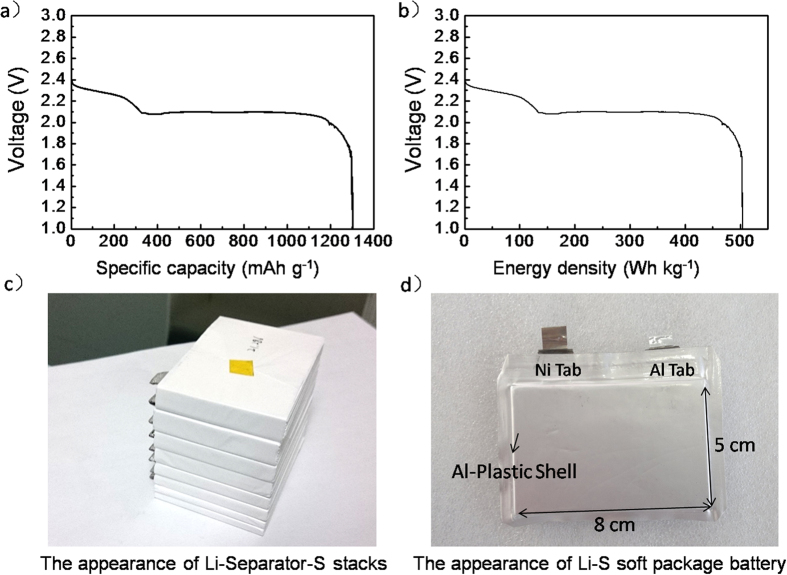
(**a**) The voltage-capacity discharge plot and (**b**) voltage-energy density plot Li-S soft package battery, (**c**) the inner and (**d**) outside appearance of the Li-S soft package battery.

**Table 1:** Characteristic property of primary batteries with high energy density.

Primary Battery type	Practical Energy density/Wh kg^−1^	Theoretical energy density/Wh kg^−1^	Application
Zn-O_2_	350–500	1086	Beacon light, radio relay station, military wireless transmitter, unmanned station, electric vehicles, etc
Li-O_2_	—	5200	—
Na-O_2_	—	1600	
Li-SO_2_	260	1170	Smart meter, highway automatic charge system, tracking system, security systems, automotive, marine, military, aerospace, information technology, radar transponders, logic program controller, radio alarm sensor, ocean buoy and remote control system. watches, pocket calculators, radio receiver, photographic equipment, etc
Li-SOCl_2_	650	1470	
Li-MnO_2_	400	1005	
Li-CF_x_	590	2180	
Li-S	430	2600	Similar application areas as the above mentioned

**Table 2:** The BET parameters of KB and MKB particles.

Parameters^a^	KB	MKB
Total surface area (m^2^g^−1^)	1349	1173
Total pore volume (cm^3^g^−1^)	2.61	3.13
Pore volume _<20_ _nm_ (cm^3^g^−1^)	1.38	1.17
Pore volume _>20_ _nm_ (cm^3^g^−1^)	1.23	1.96
Percentage of pore volume _>20_ _nm_	47%	63%

^a^calculated by BJH method from adsorption curve.
