# Supplementary material for: Lithium Sulfur Primary Battery with Super High Energy Density: Based on the Cauliflower-like Structured C/S Cathode
Source: Sci Rep. 2015 Oct 12;5:14949. doi: 10.1038/srep14949 (PMC4601074; doi:10.1038/srep14949)
Supplement: Supplementary Information [file srep14949-s1.doc]

**Lithium Sulfur Primary Battery with Super High Energy Density: Based on the Cauliflower-like Structured C/S Cathode**

**Yiwen Ma** *^a^***, Hongzhang Zhang** *^a^* ***, Baoshan Wu** *^a^***, Meiri Wang** *^a^***, Xianfeng Li** *^a^* ^b^ **and Huamin Zhang** *^a^* ^b^ *****

*^a^* Division of Energy Storage, Dalian Institute of Chemical Physics, Chinese Academy of Sciences, 457 Zhongshan Road, Dalian 116023 (P.R. China),

^b^ Collaborative Innovation Center of Chemistry for Energy Materials (iChEM), Dalian 116023 (P.R. China)

**1. The preparation of cathode materials:**

1.1. The MKB materials preparationmethods

First, 4 g KB 600 was added to 100 mL deionized water under vigorous stirring at 70 ^o^C, followed by addition of 33.34 g aqueous solution of gelatin (3 wt %) and stirred for another 8 hours. Then the mixture was dried at 60 ^o^C and crushed in a mortar to yield the KB-gelatin composite. Then the composite was transferred to a quartz boat in oven. The oven was ramped to the desired temperature using a programmed procedure: from 20 ^o^C to 600 ^o^C at a rate of 10 ^o^C min^-1^ and then to 900 ^o^C at a rate of 5 ^o^C min^-1^ and hold at 900 ^o^C for 1 h. The sample was collected and denoted as MKB.

As illustrated in Figure S1, the gelatin tender to connect the carbon particles in a “point-to-point” form, compared with the PVDF binder which tender to form a polymer network. During treating at 900 ^o^C, the gelatin would gradually be carbonated, which could still joint the carbon particles together tightly. In the contrary, the PVDF network would fall in to powder during the carbonating process.


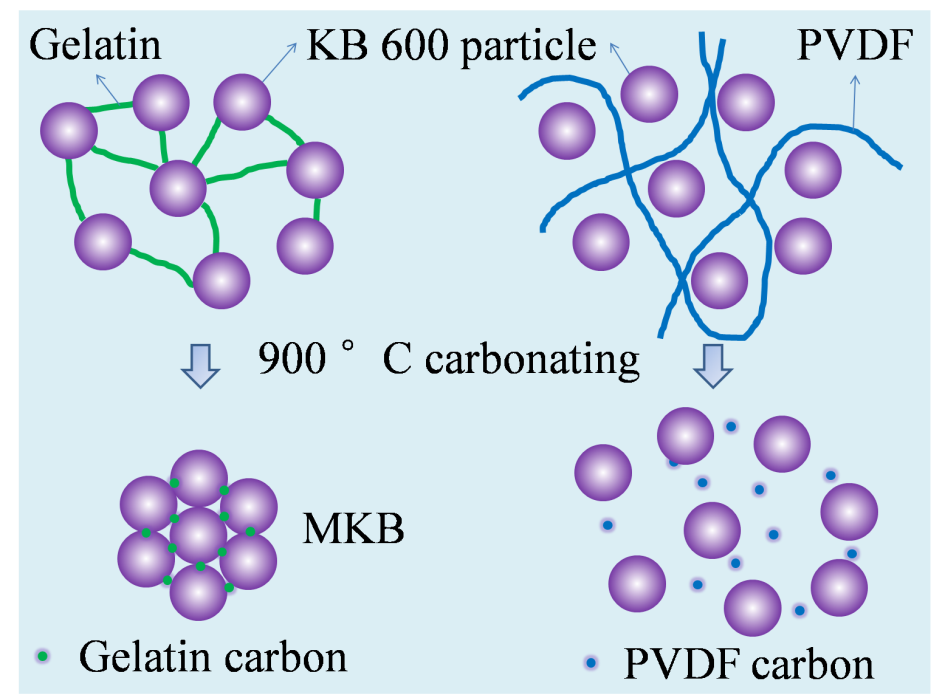


**Figure S1.** Schematic representation of different method to cohere the carbon (KB600) particles. (left) Inter-particle brides formed by gelatin. (right)The polymeric network formed by PVDF in which the particles are simply entrapped.

1.2. The C/S composite preparation:

The MKB-S composite was prepared using a melt-diffusion method, as illustrated in Figure S2. Briefly, 1.2 g MKB and 3.8 g sulfur were mixed together, and then transferred to a quartz boat in the oven. The oven was then ramped to the desired temperature using a programmed procedure: First, from 20 ^o^C to 155 ^o^C at a rate of 5 ^o^C min^-1^ and hold at 155 ^o^C for 4 h. The weight ratio of sulfur/carbon was adjusted to be equal to or less than 3:1. The obtained material was denoted as MKB-S. The KB-S and Super p-S composites were prepared the same as MKB-S.


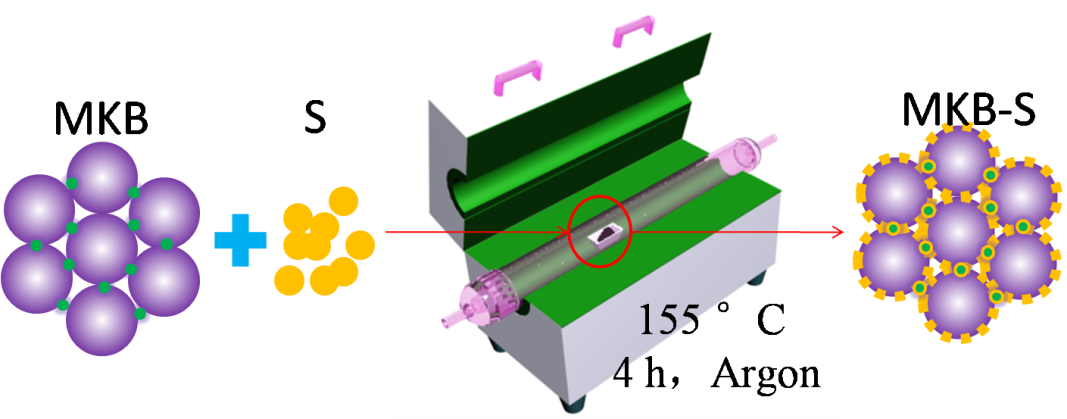


**Figure S2.** The C/S composite preparation process illustration.

1.3. The cathode preparation:

The cathode was prepared with 16 μm aluminum foil as the current collector and CMC/SBR as the binder. First, 3.8 g C/S composite was mixed with 0.08 g CMC and 10 g water. After a vigorous stirring for 4 hours, 0.3 g SBR solution (40 wt%) was added into the slurry and stir for another 4 hours. Before casting, another 5 g water was added to keep the total solid content of the slurry at 20 wt%. Then, the slurry was cast on to the aluminum foil with different thickness and dried at 70 °C over night. As for the soft package battery assembly, both sides of the aluminum were coated with C/S composite. The cathodes of KB-S and Super P-S were prepared in the same technological process. The cathode coating equipment is shown in Figure S3.


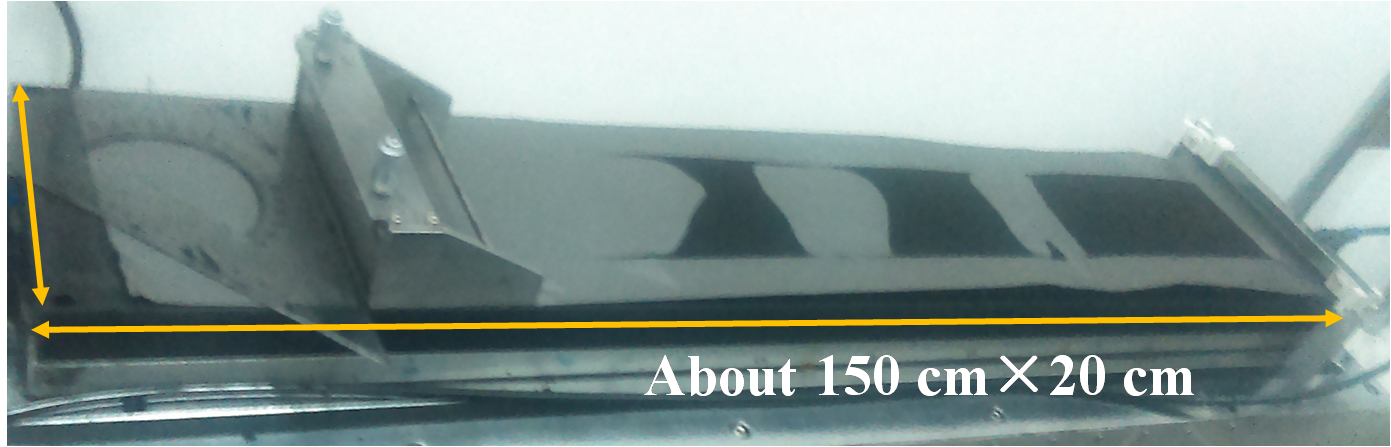


**Figure S3.** The cathode coating machine used for soft package batteries manufacture.

As for the binding strength difference of KB-S and MKB-S carbon powder on aluminum foil, in order to achieve the same adhesion quality, less binder is needed for the MKB-S particles (Figure S4). As a result, the MKB-S is preferred for practical application, for further improving the energy density and electrical conductivity of the whole cathode.


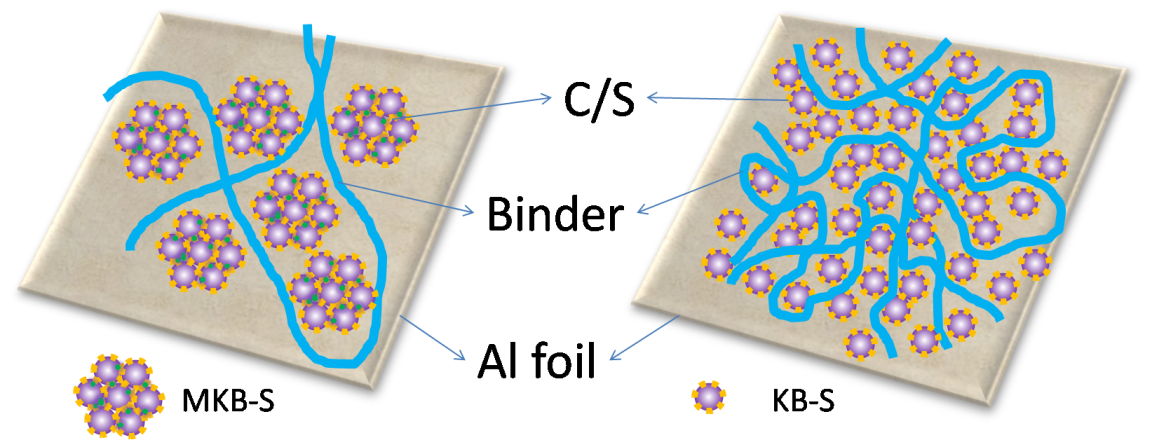


**Figure S4.** Schematic representation of binder and C/S composite on the aluminum foil: (left) inter-particle is MKB-S, (right) inter-particle is KB-S.

1.4 The battery assembly method

The as prepared cathodes were tested with both coin cells and soft package batteries. As for the coin cell assemble: first, the lithium anode with diameter of 18 μm was put into the coin 2016 cell; second, the Celgard 2325 separator with diameter of 19 μm was added on the surface of the lithium; then, the electrolyte was added onto the separator; last, the cathode with diameter of 16 μm was added onto the separator. Then the 2016 cell was sealed with machine. The electrolyte was composite of 1 M LiTFSI in DOL/DME (1:1 v/v) solution, and the electrolyte adding amount was 20 times of the sulfur content (by weight) (LiTFSI: Bis-(trifluoromethane) sulfonimide lithium, Guotai-Huarong New Chemical Materials Corp; DME:dimethoxyethane, Guotai-Huarong New Chemical Materials Corp. and DOL: 1,3-dioxolane,Guotai-Huarong New Chemical Materials Corp.).The entire coin cell assembling process was finished in the glove box filled with argon.

As for the soft package battery assemble: first, the lithium foil with the length of 2 m was spread on to a glass plate; then, the Celgard 2325 membrane with the length of 4 m was used to swamp the lithium foil; third, tens of the cathode with the size of 5 cm × 8 cm was stacked between the Celgard 2325 separators and lithium folded in the Z type; then, the electrodes stacks were pressed by hand and packed into aluminum plastics shells; last, the electrolyte was added into the aluminum plastics shells before sealed. As to be noted that, the soft package stacks were pressed tightly to ensure no more electrolyte was remained in the corners of the aluminum plastics shells.

2**. Material characterization.**

2.1 TEM morphology of the carbon and C/S composite materials

The morphology of the carbon and C/S composite materials was observed under TEM, with the samples dispersed on the copper net with alcohol. The size of the single carbon particle was measured as shown in Figure S5.


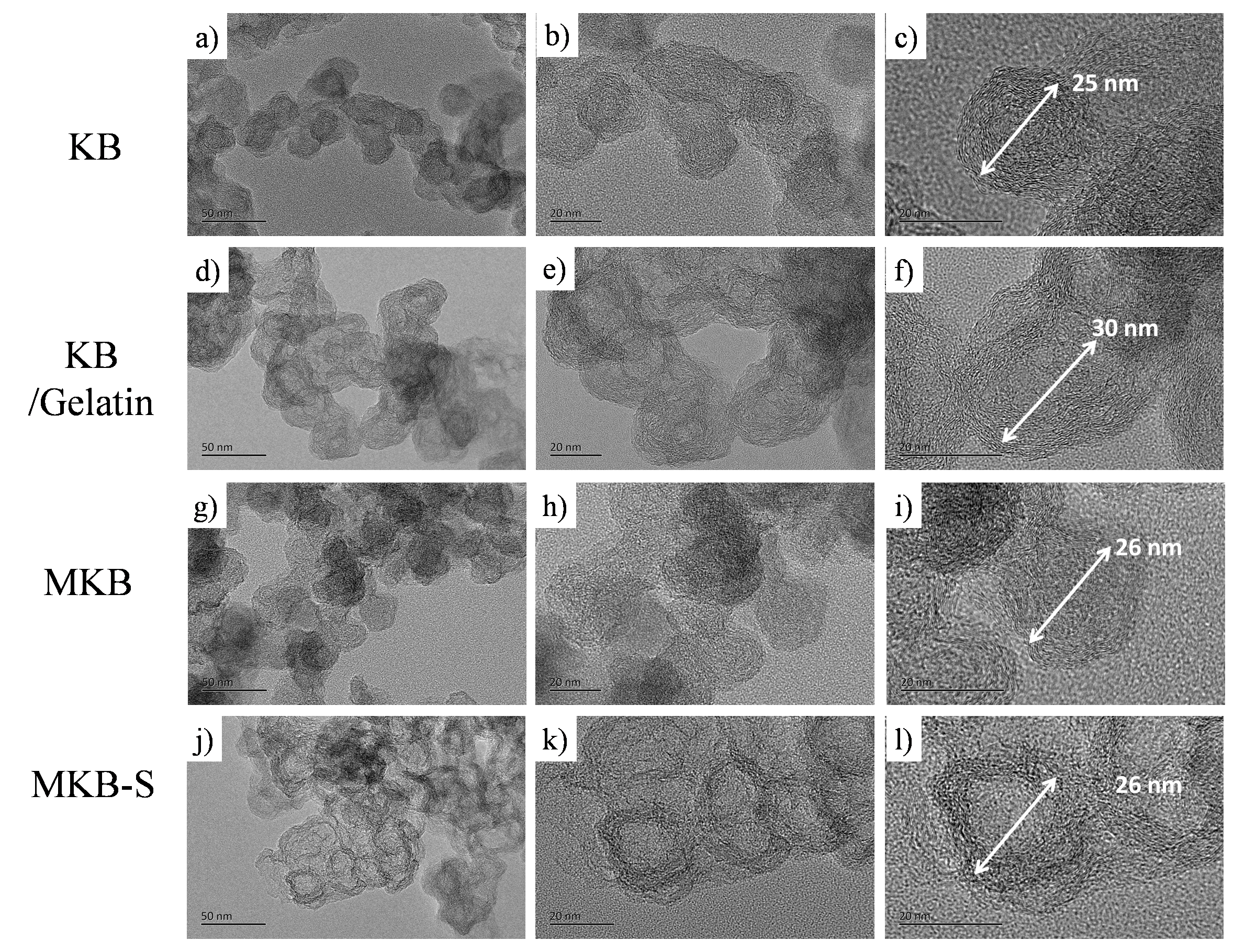


**Figure S5.** The TEM morphology of the pristine KB600 (a-c), KB600/gelatin (d-f), MKB (g-i) and MKB-S (j-l).

2.2 BET property of carbon materials

Brunauer–Emmett–Teller (BET) method was used for the surface area measurement. Barrett–Joyner–Halenda (BJH) adsorption-desorption was used for the pore analysis. Quantitative pore parameters of both KB and MKB before sulfur loading were derived from the BJH and BET methods. As shown in Figure S6a and S6b, both KB and MKB shows typical IV isotherms with a significant hysteresis loop from the N_2_ adsorption/desorption isotherm, indicating the existence of abundant mesoporous. These pores are mainly concentrated below 20 nm and over 20 nm from the pore size distribution in Figure S6c. Because of the blockage of surface pores with gelatin, the specific surface area of MKB decreased from 1349 m^2^ g^-1^ of KB to 1173 m^2^ g^-1^, together with the volume of pores below 20 nm decreasing from 1.38 to 1.17cm^3^g^-1^. However, due to the purposely formed carbon clusters structure, the volume of pores between 20 to 100 nm increased sharply from 1.23 to 1.96 cm^3^g^-1^. As a result, the total pore volume of MKB is 0.5 cm^3^g^-1^ higher than the pristine KB600, exhibiting better ability to accommodate the sulfur species. (Figure S6d)


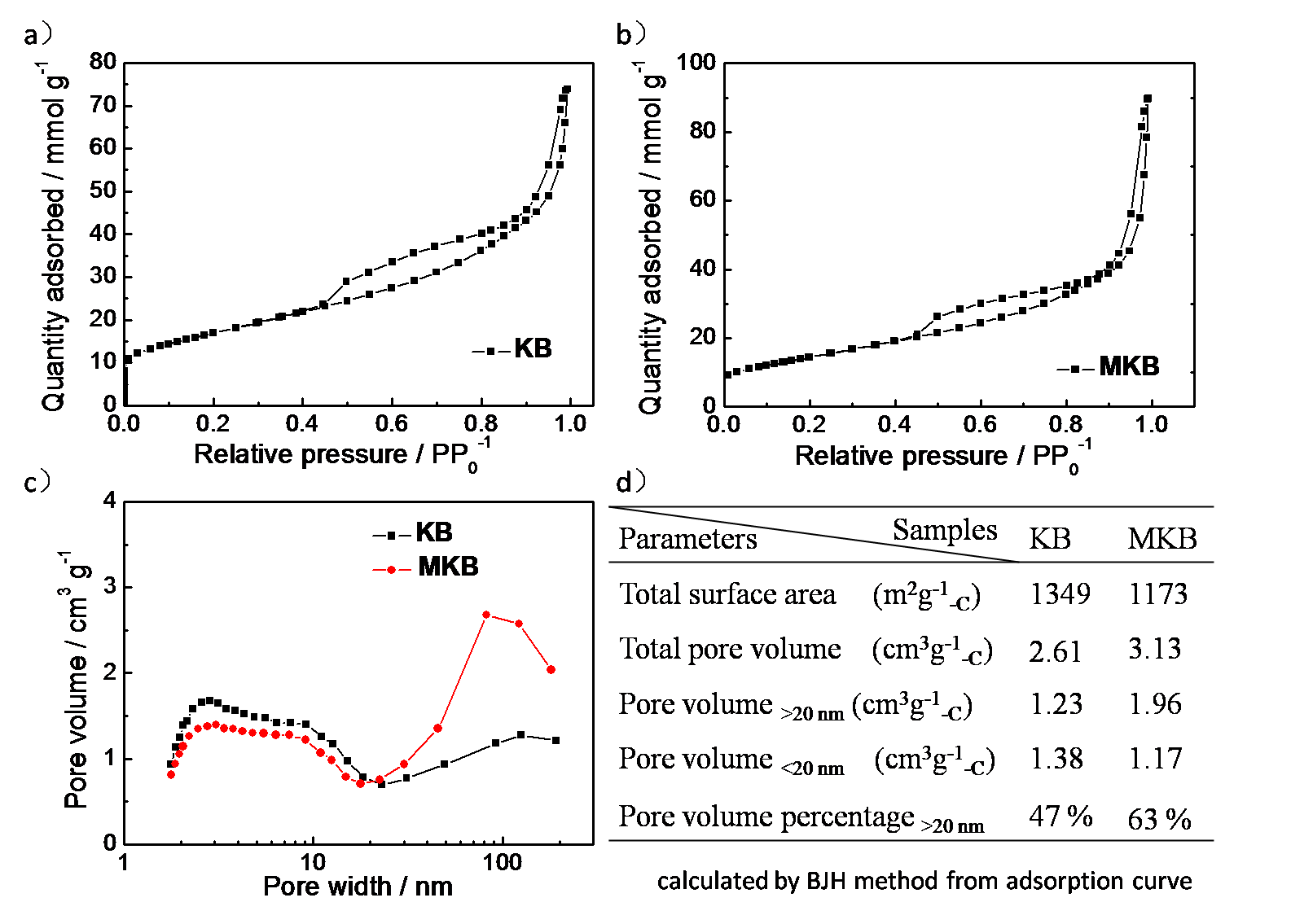


**Figure S6.** BET data of KB and MKB: (a) Nitrogen adsorption/desorption isotherms of KB, (b) Nitrogen adsorption/desorption isotherms of MKB, (c) pore size distribution curves of KB and MKB, (d) statistical data of pore volume and BET surface area.

2.3 Electrolyte uptake of cathodes

The electrolyte uptake of cathodes was tested with TEGDME as the soaking medium, for its excellent wettability with C/S composite and low volatility at room temperature. First, the cathodes (8 cm × 5 cm) were immersed in to the TEGDME for 3 hours, until the electrolyte was soaked in to the macro-, meso- and micro pores of the cathode. Then, the wet weight of the cathode was weight with an analyticalbalance. The electrolyte uptake of the cathodes was calculated according to Eq. S1. The electrolyte uptake of cathodes was illustrated in Table S1.


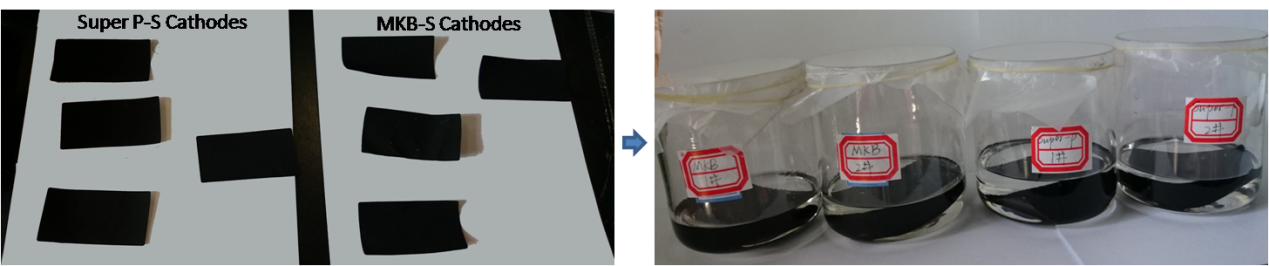


**Figure S7.** The digital photos of cathodes used for electrolyte uptake.

$\text{Electrolyte uptake}_{\text{per S mass}}\text{ =}\frac{\text{W}_{\text{wet}}\text{-W}_{\text{dry}}}{\text{m}_{\text{c/s}}}\times\frac{1}{\rho_{\text{TEGDME}}}\times100\%$ (Eq.S1)

$$\text{Electrolyte uptake}_{\text{per cathode volume}}\text{ =}\frac{\text{W}_{\text{wet}}\text{-W}_{\text{dry}}}{\text{m}_{\text{c/s}}}\times\frac{1}{\rho_{\text{TEGDME}}}\times\frac{1}{\text{V}_{\text{cathode}}}\times100\%$$

(W_wet_: the cathodes weight after soaking with TEGDME. W_dry_: the cathodes weight before soaking with TEGDME. m_c/s_: the mass of C/S composite on the cathodes. $\rho_{\text{TEGDME}}$: the density of TEGDME testedas 0.99 g cm^-3^；V_cathode_: the volume of the cathode without of aluminum)

**Table S1.** The electrolyte uptake of cathodes.

| **Electrode** | **Thickness** | **Sulfur loading** | **Electrolyte uptake** | |
| --- | --- | --- | --- | --- |
|  |  |  | Per S mass  mL mg^-1^ | Per cathode volume  mL cm^-3^ |
| **MKB-S** | 139 μm | 5.72 mg cm^-2^ | 2.27 | 3.23 |
| **Super P-S** | 161 μm | 5.62 mg cm^-2^ | 1.30 | 1.59 |
| **KB-S** | The quality of the coating was too bad for this experiment | | | |

2.4 Elements analyst of the cathodes before and after discharge

The elements on surface of the discharged cathodes were detected with EDX method. As shown in Figure S8, the both the Super P-S and the MKB-S cathodes are composited with C, S elements. The F and O elements were originated from the ether/Li-TFSI electrolyte. As shown that, the Super P-S cathode was covered with a dense layer of Li_2_S/Li_2_S_2_, while the MKB-S cathode still kept the porous structure.


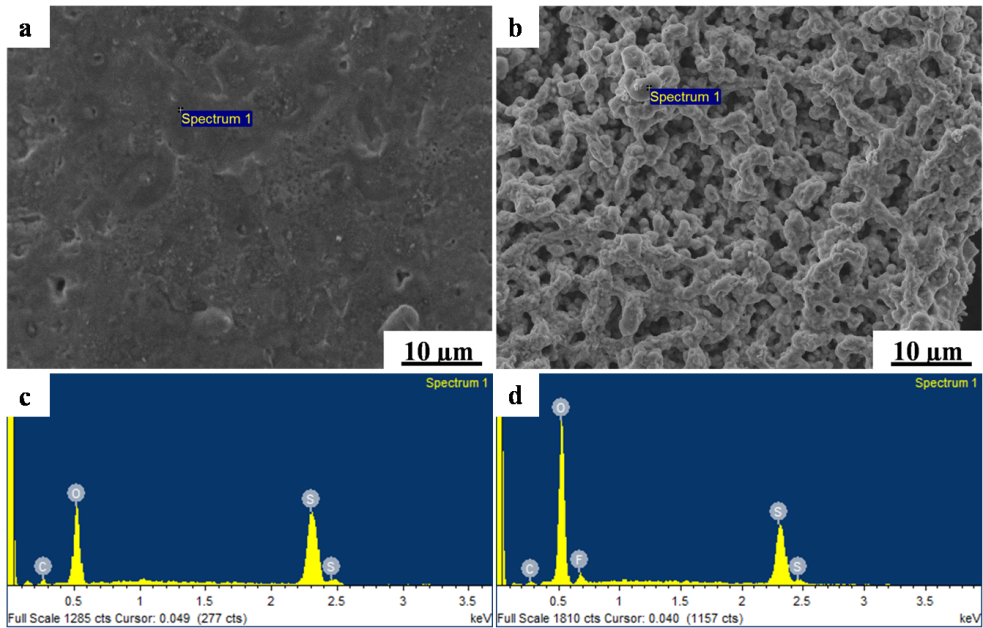


**Figure S8.** EDX of the Super P-S e (a, c) and MKB-S (b, d) cathode after discharge

2.5 The cycling performance of MKB-S cathodes

Although the MKB-S cathodes are mainly designed for primary application, its cycling stability was also carried out under 0.01C at room temperature. As shown in Figure S9, the cycle stability of MKB-S cathodes decreased with the increase of sulfur loading. Interestingly, the cathode with sulfur of 6 mg cm^-2^ could still cycle over 50 times under 0.01C.


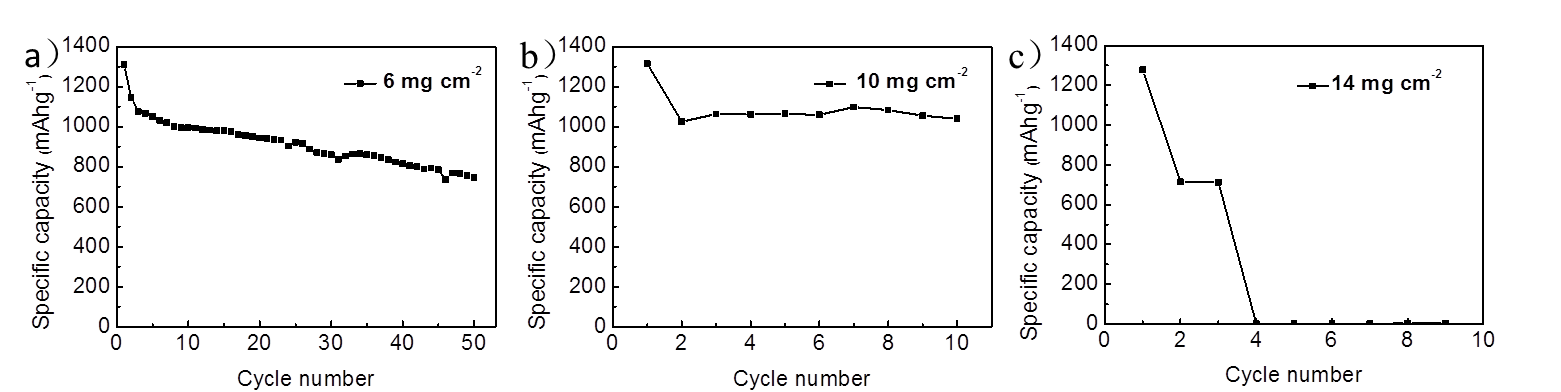


**Figure S9.** The cycling performances of MKB-S electrode with different sulfur loading: (a) 6 mg cm^-2^, (b) 10 mg cm^-2^, (c) 14 mg cm^-2^, respectively.

After 50 cycles, the Li-S coin cell was disassembled for further observation. It’s founded that the lithium was almost depleted and appeared like grey colormoss. At the same time, the separators in the dissembled battery were not transparent, which means that the separators are not thoroughly soaked with electrolyte. The exhaust of both lithium and electrolyte, caused by parasitic reaction, was deemed as the main reason of battery fail. However, the mass of lithium and electrolyte are enough for the primary application, or even several limited cycles. Besides that, the cathode still shows good mechanical strength, which further confirmed the excellent binding strength of the MKB-S as well as the abundant pore volume in the cathode (Figure S10).


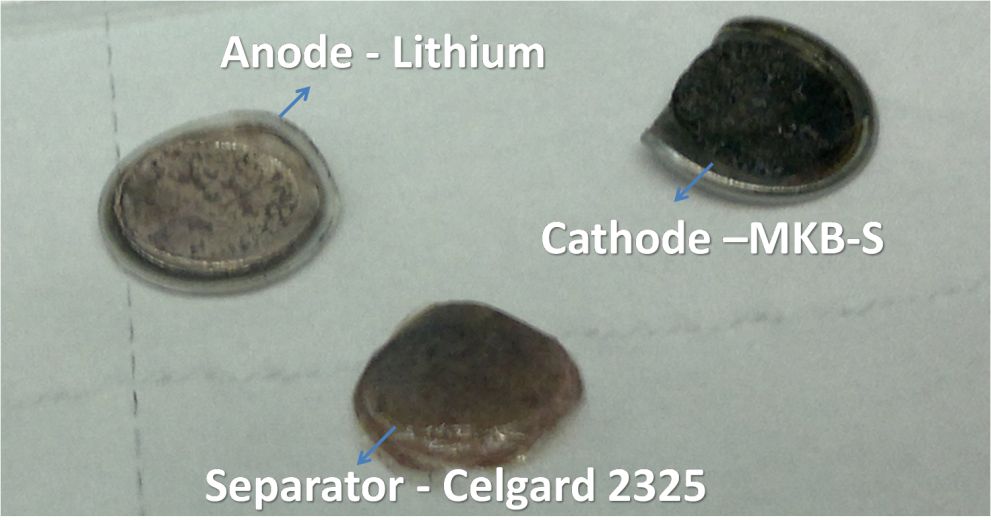


**Figure S10.** The morphology Li-S battery disassembled after cycling with 6 mg cm^-2^ sulfur loading on the cathode.

2.6 The cycling performance of MKB-S cathodes

As shown in Figure S11, the cathode could still output capacity over 800 mAh/g (s) for the 40^th^ cycle after 30 days, showing excellent shelf stability.

**
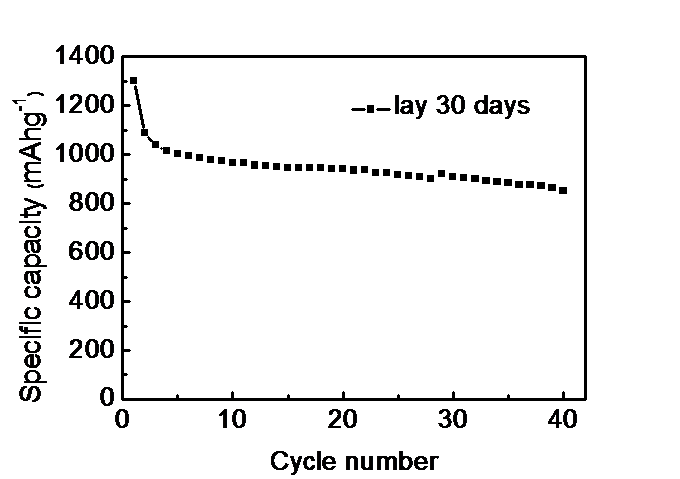
**

**Figure S11.** The cycling stability of MKB-S cathode after 30 days shelf test.

2.6 The demonstration of Li-S soft package batteries

As for the 10 Ah soft package batteries, tens of LED lights could be easily started up as shown in Figure S12. According to the relatively low cost, high energy density and good shelf life of primary Li-S battery, its development should be put into more consideration.


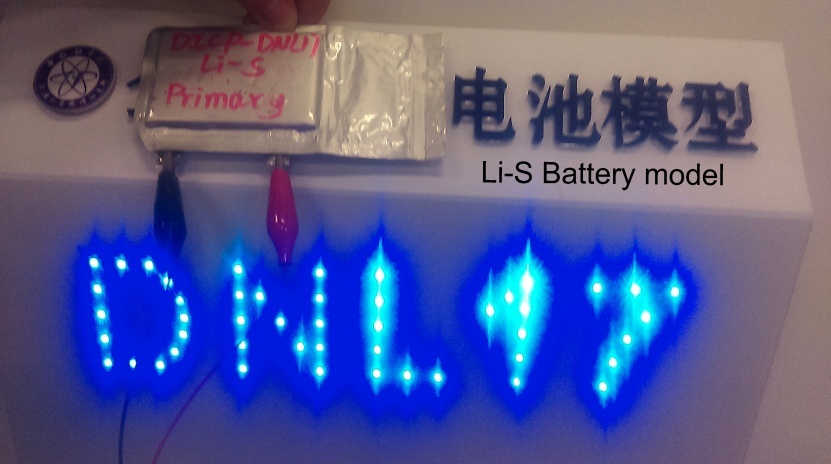


**Figure S12.** The LED light demonstration of the primary soft package Li-S battery.
